# Decoding the Architecture of the Varicella-Zoster Virus Transcriptome

**DOI:** 10.1128/mBio.01568-20

**Published:** 2020-10-06

**Authors:** Shirley E. Braspenning, Tomohiko Sadaoka, Judith Breuer, Georges M. G. M. Verjans, Werner J. D. Ouwendijk, Daniel P. Depledge

**Affiliations:** a Department of Viroscience, Erasmus MC, Rotterdam, Netherlands; b Division of Clinical Virology, Center for Infectious Diseases, Kobe University Graduate School of Medicine, Kobe, Japan; c Department of Infection and Immunity, University College London, London, United Kingdom; d Department of Medicine, New York University School of Medicine, New York, New York, USA; Princeton University

**Keywords:** direct RNA-Seq, ORF62, alphaherpesvirus, kinetics, transcriptome, varicella-zoster virus

## Abstract

Varicella-zoster virus (VZV), a double-stranded DNA virus, causes varicella, establishes lifelong latency in ganglionic neurons, and reactivates later in life to cause herpes zoster, commonly associated with chronic pain. The VZV genome is densely packed and produces multitudes of overlapping transcripts deriving from both strands. While 71 distinct open reading frames (ORFs) have thus far been experimentally defined, the full coding potential of VZV remains unknown. Here, we integrated multiple short-read RNA sequencing approaches with long-read direct RNA sequencing on RNA isolated from VZV-infected cells to provide a comprehensive reannotation of the lytic VZV transcriptome architecture. Through precise mapping of transcription start sites, splice junctions, and polyadenylation sites, we identified 136 distinct polyadenylated VZV RNAs that encode canonical ORFs, noncanonical ORFs, and ORF fusions, as well as putative noncoding RNAs (ncRNAs). Furthermore, we determined the kinetic class of all VZV transcripts and observed, unexpectedly, that transcripts encoding the ORF62 protein, previously designated *Immediate-Early*, were expressed with *Late* kinetics. Our work showcases the complexity of the VZV transcriptome and provides a comprehensive resource that will facilitate future functional studies of coding RNAs, ncRNAs, and the biological mechanisms underlying the regulation of viral transcription and translation during lytic VZV infection.

## Abstract (precis)

Transcription from herpesviral genomes, executed by the host RNA polymerase II and regulated by viral proteins, results in coordinated viral gene expression to efficiently produce infectious progeny. However, the complete coding potential and regulation of viral gene expression remain ill-defined for the human alphaherpesvirus varicella-zoster virus (VZV), causative agent of both varicella and herpes zoster. Here, we present a comprehensive overview of the VZV transcriptome and the kinetic class of all identified viral transcripts, using two virus strains and two biologically relevant cell types. Additionally, our data provide an overview of how VZV diversifies its transcription from one of the smallest herpesviral genomes. Unexpectedly, the transcript encoding the major viral transactivator protein (pORF62) was expressed with *Late* kinetics, whereas orthologous transcripts in other alphaherpesviruses are typically expressed during the immediate early phase. Therefore, our work both establishes the architecture of the VZV transcriptome and provides insight into regulation of alphaherpesvirus gene expression.

## INTRODUCTION

Varicella-zoster virus (VZV) is a ubiquitous human alphaherpesvirus and causative agent of both varicella (chickenpox) and herpes zoster (HZ or shingles) (1). Varicella results from primary VZV infection and leads to the establishment of a lifelong latent infection in sensory neurons of the trigeminal and dorsal root ganglia (2, 3). In one-third of infected individuals, VZV reactivates from latency later in life to cause HZ (1). Whereas varicella is generally experienced as a benign childhood disease, HZ is frequently associated with difficult-to-treat chronic pain (postherpetic neuralgia) (4, 5). Despite the recent availability of the highly effective HZ subunit vaccine (Shingrix), the health and societal burden of HZ and its complications remain high due to adverse side effects, the high cost of the vaccines, and changing demographics (6).

The 125-kb double-stranded DNA (dsDNA) genome of VZV, first sequenced in 1986, encodes at least 71 unique open reading frames (ORFs) that are expressed during lytic infection (7, 8). The current annotation of the VZV genome largely relies on both *in silico* ORF predictions and homologous ORFs in the closely related human alphaherpesvirus herpes simplex virus 1 (HSV-1) (8). For most VZV ORFs, the boundaries of transcription are not accurately determined, meaning that transcription start sites and polyadenylation sites are poorly resolved. Moreover, *in silico* ORF prediction does not account for the possibility of spliced transcripts. Indeed, we recently applied ultradeep short-read RNA sequencing to define the latent viral transcriptome and discovered the spliced VZV latency-associated transcript (VLT) (9).

The compact nature of viral genomes, combined with their ability to encode overlapping RNAs, presents a significant challenge to studies that rely on interrogating genome sequences or that use viral mutants to probe the function(s) of viral proteins. Here, a single point mutation or frameshift may impact multiple distinct RNAs at the same time, a factor that may be further confounded by inaccurate or missing transcript annotations. The reannotation of the human cytomegalovirus (HCMV) transcriptional and translational landscape and subsequent refinements of HSV-1, Kaposi’s sarcoma-associated herpesvirus (KSHV), and human herpesvirus 6 (HHV6) transcriptome architectures have all demonstrated that herpesviruses exhibit a complex transcriptional pattern of alternative splicing, opposing transcription, read-through transcription, fusion transcripts, 5′ untranslated region (5′ UTR) and 3′ UTR variations, and previously unidentified noncoding RNAs (ncRNAs) (10–17). Indeed, recent cDNA-based long-read sequencing also indicated that the lytic VZV transcriptome is substantially more complex than previously recognized (18).

By analogy to other herpesviruses and limited experimental data (19, 20), VZV transcripts and their encoded proteins have been divided into three kinetic classes: immediate early (*IE*), early (*E*), and late (*L*). Expression of *E* and *L* transcripts is considered dependent on viral proteins of the preceding kinetic classes, while expression of *IE* transcripts occurs in the absence of viral protein synthesis (21). Prior studies have defined four VZV proteins encoded by ORF4, ORF61, ORF62, and ORF63 as being transcriptional regulators that initiate lytic transcript expression (22–26), whose corresponding transcripts have been classified as *IE* by analogy to their HSV-1 orthologues. *L* transcripts, such as _lyt_VLT, the lytic isoform of VLT, are expressed either at very low levels prior to or exclusively after viral DNA replication has commenced (9). However, the species specificity and highly cell-associated nature of VZV *in vitro* have hampered detailed analysis of VZV transcription. Improved protocols to obtain cell-free VZV and mass spectrometry have provided some insight into the temporal pattern of viral protein expression (27) but lack sensitivity compared to RNA sequencing and do not provide information on viral transcription.

To address this, we have decoded the architecture of the lytic VZV transcriptome in both human epithelial cells and neurons while contrasting discrete VZV strains. We subsequently established the kinetic class of all lytic viral transcripts and integrated these results to provide a comprehensive overview of the complexity and structure of the lytic VZV transcriptome as a rich resource that will enhance future functional studies of VZV biology.

## RESULTS

### Decoding the complexity of lytic VZV gene expression.

**FIG 1:**
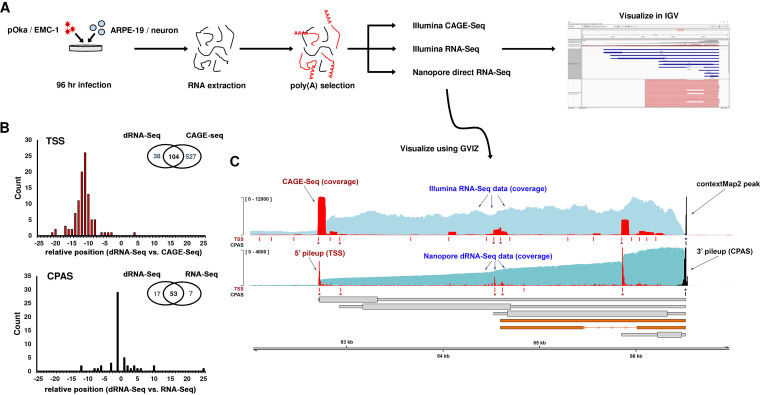
Decoding the complexity of the lytic VZV transcriptome. (A) Experimental strategy: ARPE-19 cells and hESC-derived neurons were infected with VZV EMC-1 (clade 1) or pOka (clade 2) for 96 h. Total RNA was extracted, and poly(A) fraction was isolated for sequencing by Illumina CAGE-Seq, Illumina RNA-Seq, and/or Nanopore dRNA-Seq. Sequence data were aligned against the VZV strain Dumas reference genome (GenBank accession no. NC_001348.1) and visualized using the Integrative Genomics Viewer (IGV) (32) and GVIZ (74). (B) Transcription start sites (TSS) as well as cleavage and polyadenylation sites (CPAS) were identified in Nanopore and Illumina data sets. Histograms show the distances observed between Nanopore and Illumina predictions, while inset Venn diagrams indicate the numbers of sites identified and their conservation between data sets. (C) Integration of Illumina RNA-Seq, Illumina CAGE-Seq, and Nanopore dRNA-Seq data sets. Coverage plots for Illumina RNA-Seq (light blue), CAGE-Seq (red), and Nanopore dRNA-Seq (teal) are integrated with pileup data that maps TSS (red) and CPAS (black). Rows denoted by TSS and CPAS indicate positions of TSS and CPAS identified using HOMER software (77) for Nanopore dRNA-Seq and Illumina CAGE-Seq data, and ContextMap2 software (71) for Illumina RNA-Seq data. TSS and CPAS that are conserved between sequencing technologies are indicated with asterisks, and only these are used to define the boundaries of newly annotated transcripts. RNA structures (gray) are inferred from these conserved sites. Wide and thin boxes indicate canonical coding sequence (CDS) domains and untranslated regions (UTRs), respectively. Newly identified RNAs (orange) are shown without predicted CDS domains.

Standard methods for annotating viral transcriptomes require the integration of multiple types of Illumina RNA sequencing (RNA-Seq) data to identify transcription start sites (TSS), cleavage and polyadenylation sites (CPAS), splice sites, and transcript structures. The last is particularly challenging to infer using conventional short-read sequencing approaches (28). In contrast, direct RNA sequencing (dRNA-Seq) using Nanopore arrays offers the potential to capture all these distinct data points in a single sequencing run (10, 29). To examine the structure of the lytic VZV transcriptome, ARPE-19 cells were infected with the VZV pOka wild-type strain and total RNA was extracted at 96 h post-infection (hpi) (Fig. 1A). The 96-hpi time point was chosen to maximize the diversity of VZV transcripts likely to be present. Sequencing of the polyadenylated RNA fraction was performed using both RNA-Seq and dRNA-Seq (Fig. 1A). Whereas short reads generated by standard Illumina RNA-Seq are not amenable for accurate isoform reconstruction in complex reads, they provide higher sequencing depth and detection of CPAS and, crucially, enable splice-site correction of dRNA-Seq reads via the junction-polishing package in the software FLAIR (30). In contrast, dRNA-Seq can sequence full-length RNAs and provides critical information on the presence of discrete RNA isoforms with regions of overlap, while also allow mapping of TSS and CPAS. Finally, we performed Illumina Cap Analysis Gene Expression Sequencing (CAGE-Seq) (31) to map TSS by an orthologous approach (Fig. 1A).

TSS and CPAS estimates provided by dRNA-Seq data closely overlapped those derived from our Illumina approaches (Fig. 1B). TSS sensitivity was nearly 4-fold higher in CAGE-Seq data sets than with dRNA-Seq, with effectively all TSS uniquely found by CAGE-Seq being low abundance—likely reflecting artifacts derived from RNA processing (i.e., recapping of cleaved RNA) or a generalized dysregulation of transcription initiation accompanying the late stages of a viral infection. A total of 104 TSS overlapped between the dRNA-Seq and CAGE-Seq data sets, all of which were the most abundant TSS in both data sets (see Table S1 in the supplemental material). Importantly, dRNA-Seq TSS estimates were located up to 20 nucleotides (nt) downstream (median 11 nt) of TSS derived via CAGE-Seq (Fig. 1B). This difference is best explained by the presence of low-quality ends of dRNA-Seq reads that are not aligned when using local alignment strategies. CPAS sensitivity was higher in dRNA-Seq than RNA-Seq data sets (70 versus 60 sites) with 53 sites detected by both approaches (Table S1). CPAS estimates provided by dRNA-Seq aligned closely with those derived from RNA-Seq data (median 1-nt difference, Fig. 1B) due to the 3′→5′ direction of dRNA-Seq. Finally, we reconstructed the VZV transcriptome using TSS and CPAS to define transcript structures followed by visual confirmation of read data to identify splice sites, define alternatively spliced transcripts, and examine read-through transcription (Fig. 1C). For a given transcript to be included in our final annotation, we required the specified TSS and CPAS to be present in both CAGE-Seq and dRNA-Seq data sets (Table S1). We further required that the transcript could be verified by eye through inspection of sequence read alignments in BED files (available at https://github.com/DepledgeLab/vzv-2.0) using IGV (32) and that splice junctions (Table S2) were reported in both the RNA-Seq and dRNA-Seq data sets. This is exemplified in Fig. 1C where five TSS (indicated by asterisks) were identified by both dRNA-Seq and CAGE-Seq while only a single CPAS was identified in the RNA-Seq and dRNA-Seq data. This leads us to initially annotating five distinct transcripts (each with a unique TSS and common CPAS), while further inspection of the sequence read alignments in IGV confirmed the presence of both an unspliced and spliced transcript sharing the same TSS and CPAS (shown in orange in Fig. 1C), resulting in six distinct transcripts in total.

10.1128/mBio.01568-20.5 TABLE S1 TSS and CPAS identified within the VZV genome. Download Table S1, XLSX file, 0.05 MB. Copyright © 2020 Braspenning et al. 2020 Braspenning et al. This content is distributed under the terms of the Creative Commons Attribution 4.0 International license.

10.1128/mBio.01568-20.6 TABLE S2 Splice sites utilized during VZV transcription. Download Table S2, XLSX file, 0.01 MB. Copyright © 2020 Braspenning et al. 2020 Braspenning et al. This content is distributed under the terms of the Creative Commons Attribution 4.0 International license.

### Reannotation of the VZV transcriptome reveals alternative transcript isoforms and putative noncoding RNAs.

**FIG 2:**
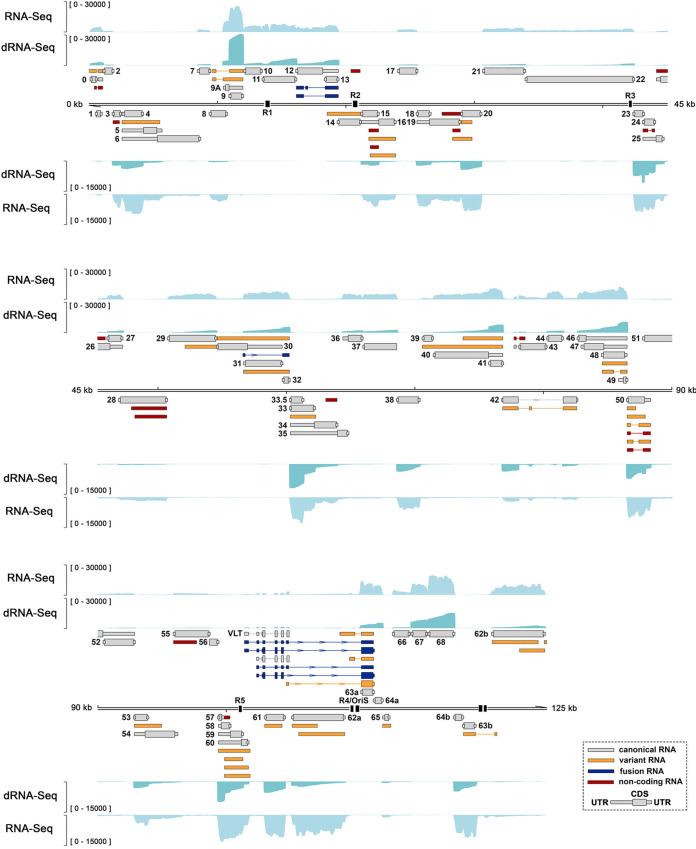
Reannotation of the lytic VZV transcriptome reveals novel viral transcript isoforms, fusion transcripts, and putative noncoding RNAs. The reannotated lytic VZV transcriptome includes 71 transcripts encoding canonical ORFs with their UTRs defined (gray), 39 alternative isoforms of existing RNAs (orange), 7 fusion RNAs (dark blue), and 17 putative polyadenylated ncRNAs (red), the last predicted using CPC 2.0 (36). Wide and thin boxes indicate canonical CDS domains and UTRs, respectively. Absence of CDS regions indicates that the respective VZV RNA has an uncertain coding potential. Illumina RNA-Seq (light blue) and Nanopore dRNA-Seq (teal) coverage plots are derived from ARPE-19 cells lytically infected with VZV strain pOka for 96 h. *y* axis values indicate the maximum read depth of that track. See also Fig. S1 and S2. Reiterative repeat regions R1 to R5 and both copies of the OriS are shown as black boxes embedded in the genome track.

**FIG 3:**
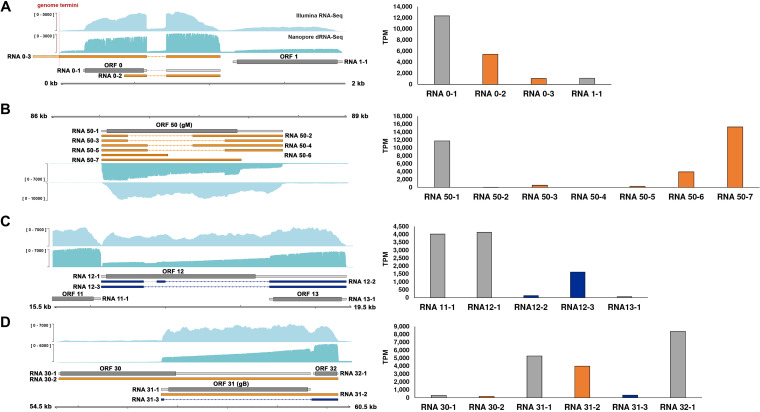
Examples of specific variant and fusion viral RNAs expressed during lytic VZV infection. (A to D) Examples of variant transcripts encoding ORF0 (A) and ORF50 (B) and novel fusion transcripts encoding (parts of) ORF12 and ORF13 (C) and ORF31 and ORF32 (D). Illumina RNA-Seq (light blue) and Nanopore dRNA-Seq (teal) coverage plots are derived from ARPE-19 cells lytically infected with VZV strain pOka for 96 h. Fusion RNAs are shown in dark blue, with variant RNAs shown in orange and canonical RNAs in gray. The dashed red line in panel A indicates the position of the genome termini spanned by RNA 0 to 3. (A to D) Genome coordinates are shown, while strand is indicated by placement of the genome track (below, top strand; above, bottom strand). *y* axis values indicate the maximum read depth of that track. For graphs shown on the right, the *y* axis denotes transcript per million (TPM) counts.

In VZV pOka-infected ARPE-19 cells, we identified 136 distinct VZV RNAs that were readily detectable at 96 hpi. Along with defining the UTRs of 96 RNAs encoding the 71 canonical VZV ORFs, we also identified 40 additional RNAs (Fig. 2 and Table S3). To reduce confusion, we numbered all RNAs according to the respective canonical ORFs and delineated transcript isoforms encoding at least part of the same ORF by number. For instance, three transcripts are transcribed from the ORF0 locus and these are here referred to as VZV RNA 0-1, 0-2, and 0-3. The identified RNAs included transcripts encoding 5′ extended ORFs, 5′ truncated ORFs, 3′ extended ORFs, 3′ truncated ORFs, internally spliced variants, and putative noncoding RNAs (ncRNAs). Importantly, our study confirmed several previously described RNA isoforms such as a genome-terminus-spanning RNA that encodes ORF0 (RNA 0-3) (33) and identified an additional 5′ truncated ORF0 RNA of unknown function (RNA 0-2) (Fig. 3A). Similarly, extensive low-level internal splicing of ORF50 has previously been reported (34) (RNAs 50-1 to 50-5) and was similarly observed here, supplemented by two additional 5′ truncated isoforms (RNAs 50-6 and 50-7) expressed at relatively high abundance (Fig. 3B). Examples of previously undocumented RNAs include two new spliced ORF9 transcript isoforms and two internally spliced transcript isoforms containing N-terminal ORF24 and ORF48 coding sequence (CDS) domains that are spliced into novel C-terminal domains (Fig. 2). Finally, we confirmed expression of transcripts encoding the novel ORF9 and ORF48 variants in VZV-infected ARPE-19 cells by RT-PCR (Fig. S1A and G and Fig. S1B and H).

10.1128/mBio.01568-20.1 FIG S1 Confirmation of selected novel viral RNAs expressed during lytic VZV infection. The structure and location of selected VZV transcripts are shown alongside gel images confirming their expression. (A to F) Illumina RNA-Seq (light blue) and Nanopore dRNA-Seq (teal) coverage plots are derived from 96-h cell-associated infections of ARPE-19 epithelial cells. Transcript colors denote their status as canonical (gray), variant (orange), fusion (blue), or putatively noncoding (red). *y* axis values indicate the maximum read depth of that track. (G to L) RT-PCR was performed on RNA extracted from ARPE-19 cells infected with cell-free VZV EMC-1 for 72 h. RT+/RT-, reverse transcriptase added or omitted during cDNA synthesis. All numbered bands were confirmed by Sanger sequencing of the RT-PCR product. Primer sequences are indicated in Table S5. (G) RNA 9A/9 variants: pair A, ORF9var_Fw–ORF9var_Rv; #1, unspliced; #2, RNA 9-2; #3, RNA 9A-2; pair B, ORF9var_Fw–ORF9var1_spliceRv; #4, RNA 9-2; pair C, ORF9var_Fw–ORF9var2_splice_Rv; #5, RNA 9A-2. (H) RNA 48 variants: pair A, ORF48_Fw–ORF48_Rv; #6, RNA 48-1/48-2; #7, RNA 48-3; pair B, ORF48_Fw–ORF48_spliceRv; #8, RNA 48-3. (I) RNA 12-13 fusion RNA: ORF12-13_Fw–ORF12-13_Rv; #9, RNA 12-3; #10, RNA 12-2. (J) RNA 31-32 fusion RNA: pair A, ORF31-32_Fw–ORF31-32_Rv_#1; #11, RNA 31-3; pair B, ORF31-32_Fw–ORF31-32_Rv_#2; #12, RNA 31-3. (K) RNA 13.5-1: ORF13-5_Fw–ORF13-5_Rv, #13. (L) RNA 43 variants: VZV_ORF43A_Fw–ORF43.5_Rv, RNA 43-2; #14, unspliced RNA 43-1/RNA 43-2; #15, spliced RNA 43-1/RNA 43-2. (M) Northern blot analysis was performed on RNA extracted from ARPE-19 cells infected with cell-associated VZV EMC-1 or mock infected for 72 h to detect RNA 64 variants. Band #16 indicates the polycistronic RNA 63-64 precursor RNA, and bands #17 and #18 indicate RNA 64-1 and a low-abundant alternative RNA 64 isoform, respectively. Download FIG S1, TIF file, 1.0 MB. Copyright © 2020 Braspenning et al. 2020 Braspenning et al. This content is distributed under the terms of the Creative Commons Attribution 4.0 International license.

10.1128/mBio.01568-20.7 TABLE S3 The complete VZV reannotation. Download Table S3, XLSX file, 0.04 MB. Copyright © 2020 Braspenning et al. 2020 Braspenning et al. This content is distributed under the terms of the Creative Commons Attribution 4.0 International license.

10.1128/mBio.01568-20.2 FIG S2 Functional analysis of VZV RNA 43-2. (A to E) ARPE-19 cell lines stably expressing empty pcDNA3.1 (black; empty) or RNA 43-2 (light blue) were generated. Squares, circles, and triangles represent the 3 independently generated stable ARPE-19 cell lines. (A) PCR confirmation of DNA encoding RNA 43-2 in empty pcDNA3.1 or RNA 43-2 stable cell lines. (B) RT-PCR confirmation of RNA 43-2 expression from RNA 43-2 stable cell lines, RT+/RT-, reverse transcriptase added or omitted during cDNA synthesis. (C to E) Cells were infected with cell-free VZV EMC-1. (C) Percentage of VZV-infected cells (glycoprotein E [gE] positive) at 48 hpi or 72 hpi determined by flow cytometry. *P = *0.34 and *P = *0.24 by unpaired Student’s *t* test. (D) Relative plaque size (normalized to empty pcDNA3.1-transfected cell lines) at 72 hpi. *P* = 0.490 by unpaired Student’s *t* test. (E) Relative expression of RNA 43-1 and RNA 62-1 at 24 hpi*. P = *0.085 and *P = *0.449 by unpaired Student’s *t* test. (F) Relative expression levels of RNA 43-1 and RNA 43-2 in cell-cycle-synchronized VZV-infected cells after 12-h cycloheximide, 24-h mock, or 24-h phosphonoacetic acid (PAA) treatment. (G) Relative expression levels of RNA 43-1 and RNA 43-2 in cell-cycle-synchronized VZV-infected cells at the indicated time points postinfection. All expression levels were normalized to β-actin transcripts. Download FIG S2, TIF file, 1.2 MB. Copyright © 2020 Braspenning et al. 2020 Braspenning et al. This content is distributed under the terms of the Creative Commons Attribution 4.0 International license.

Fusion transcripts combine sequences from two or more distinct canonical viral transcripts and most likely result from transcription termination occurring at an alternative CPAS downstream of the canonical CPAS, followed by internal splicing (10). The resulting fusion transcripts are predicted to encode new proteins that contain fused domains from two or more distinct protein products. We identified seven VZV fusion transcripts in total. Four of these contain distinct fusions of transcripts encoding pVLT and pORF63 (Figure 2, Table S3). Internal splicing of RNA 12-2 and RNA 12-3 transcripts yields two distinct splice variants that fuse parts of ORF12 and ORF13 CDS domains (Fig. 3C). Additionally, we observed transcripts that fused the 5′ UTR of ORF31 transcripts to ORF32 transcripts, resulting in a transcript encoding pORF32 with an alternative 5′ UTR (Fig. 3D). We confirmed expression of transcripts containing the ORF12-ORF13 and ORF31-ORF32 fusions in VZV-infected ARPE-19 cells by RT-PCR (Fig. S1C and I and Fig. S1D and J).

Additionally, we discovered two novel polyadenylated VZV transcripts: RNA 13.5-1 and RNA 43-2. RNA 13.5-1 is 634 nt long, encodes two putative CDS domains (88 and 55 amino acids [aa]), and is positioned antisense to the RNA 14-1 (encoding pORF14). Like RNA 14-1, RNA 13.5-1 stretches across the R2 reiterative region, a short-repeat region that exhibits length variations between viral strains and within viral populations and thus leads to length variations in the encoded transcripts (35) (Fig. 2 and Fig. S1E). We also identified a highly expressed 3′ truncated RNA (RNA 43-2) that overlaps the 5′ end of RNA 43-1 (encoding pORF43) (Fig. 2 and Fig. S1F). RNA 43-2 is a spliced 590-nt transcript that encodes only a short CDS domain (21 aa). Expression of both RNA 13.5-1 and RNA 43-2 in VZV-infected ARPE-19 cells was confirmed by RT-PCR (Fig. S1E and K and Fig. S1F and L)

Finally, we used an *in silico* approach to predict the coding potential of all 136 polyadenylated VZV RNAs (Table S3). The Coding Potential Calculator version 2 algorithm (CPC 2.0) (36) calculates the coding probability of a transcript based on its length, isoelectric point, and Fickett score of the longest CDS encoded. Of the 96 VZV RNAs encoding the 71 canonical ORFs, 89 were assigned a coding probability exceeding 90%, with only two canonical RNAs—encoding the two smallest VZV proteins, pORF49 (81 aa) (37) and pORF57 (71 aa) (38)—incorrectly predicted to be a noncoding transcript (Table S3). Of the 40 VZV RNAs encoding noncanonical products, 17 were predicted to be noncoding (Table S3), including two novel transcripts: RNA 13.5-1 (7%) and RNA 43-2 (13%). Notably, only RNA 43-2 was expressed to relatively high levels as the 37th most abundant transcript at 24 hpi and 41st most abundant at 96 hpi (out of 136 VZV transcripts).

We next validated a selection of the reported transcript variants through comparative analysis of our sequencing-derived data against Northern blotting approaches used in prior studies. For instance, prior Northern blot analysis of the VZV RNA 50 locus (34) identified at least six discrete bands (2,100, 1,800, 1,400, 1,200, 1,000, and 900 nt), five of which corresponded to six transcripts observed in our data set (1,825, 1,400, 1,362, 1,162, 1,039, and 839 nt). We note that no annotated transcript in our data matched the observed 2,100-nt band, nor was any band observed that matched our 663-nt transcript variant. Similarly, Northern blotting of the VZV RNA 9 locus (39) demonstrated the presence of at least three bands, sized approximately 2,400, 1,600, and 1,100 nt which correspond with RNA 9-1 (1,030 nt), RNA 9A-1 (1,530 nt), and the unspliced precursors for RNA 9-2 and 9-3 (2,373 nt). Note that the presence of mature spliced RNA 9-2 (1,556 nt) and 9-3 (1,814 nt) was confounded by their size relative to RNA 9A-1. Finally, we performed Northern blotting to confirm the expression of the 698-nt RNA 64-1 and 1,653-nt polycistronic RNA 63-64, as well as a low-abundance ±400-nt RNA 64 variant that was detected, but not sufficiently evidenced for including in the annotation, in our data sets (Fig. S1M).

### The lytic VZV transcriptome is not influenced by viral strain or cell type.

**FIG 4:**
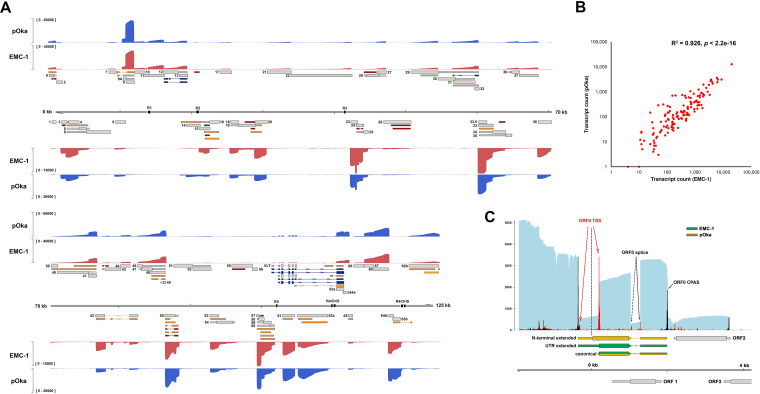
VZV transcriptome diversity is consistent between viral strains. (A) Coverage plots derived from Nanopore dRNA-Seq of VZV strain pOka (blue)- and VZV strain EMC-1 (red)-infected ARPE-19 cells at 96 hpi. Note that while relative and absolute abundances of distinct VZV RNAs differ between strains, all RNAs included in our annotation are represented. *y* axis denotes coverage range. Canonical (gray), alternative (orange), fusion (dark blue), and putative (red) ncRNAs are shown with canonical CDS regions indicated by wide boxes and UTRs shown as thin boxes. The absence of a CDS region indicates that an RNA has uncertain coding potential. The reiterative repeat regions R1 to R5 and both copies of the OriS are shown as black boxes embedded in the genome track. (B) The scatterplot shows the correlation between VZV transcript abundance in VZV EMC-1-infected and VZV pOka-infected ARPE-19 cells. Pearson *R*^2^ and *P* value are indicated. (C) Identical TSS and CPAS were detected in ARPE-19 cells infected with cell-free VZV pOka (VZV clade 2; yellow) or EMC-1 (VZV clade 1; green) at 96 hpi, yielding two ORF0 RNA isoforms. However, due to nucleotide variability between the two strains, VZV pOka RNA 0-1 includes a larger CDS and can encode an alternative ORF0 protein variant, compared to VZV EMC-1. Nanopore dRNA-Seq (teal) coverage plots are shown. *y* axis values indicate the maximum read depth of that specific track. CDS regions and UTRs are shown as wide and thin boxes, respectively.

VZV genome sequences are highly conserved (40), suggesting that strain-specific differences in coding capacity are likely minimal. To test this hypothesis, we infected ARPE-19 cells with VZV EMC-1 for 96 h and sequenced the poly(A) fraction of RNA by dRNA-Seq (Table S4). This enabled a comparative analysis of data sets obtained from ARPE-19 cells lytically infected with either VZV pOka or EMC-1 to determine whether either strain encodes unique transcripts (Fig. 4). No such transcripts were identified at the RNA level, although we note that nucleotide level changes may still impact encoded proteins—as is exemplified by the N-terminal extended pORF0 uniquely present in pOka (Fig. 4C).

10.1128/mBio.01568-20.8 TABLE S4 Overview of sequencing data sets. Download Table S4, XLSX file, 0.01 MB. Copyright © 2020 Braspenning et al. 2020 Braspenning et al. This content is distributed under the terms of the Creative Commons Attribution 4.0 International license.

**FIG 5:**
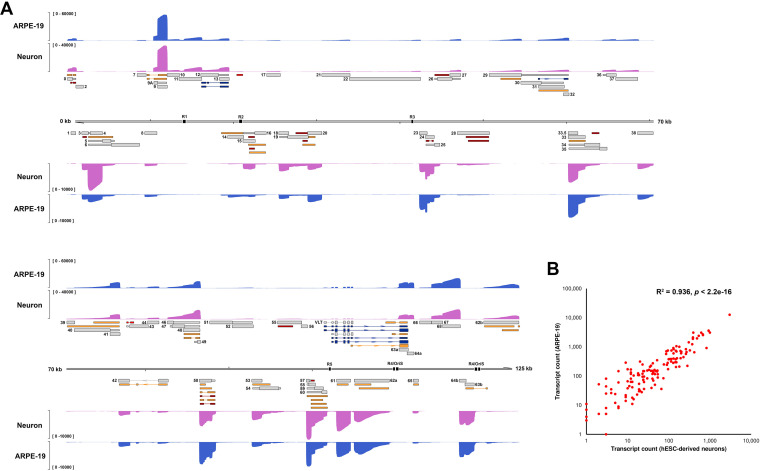
VZV transcriptome diversity is consistent between infected cell types. (A) Coverage plots derived from Nanopore dRNA-Seq of VZV pOka-infected ARPE-19 cells (blue) and hESC-derived neurons (purple). RNA was collected at 96 hpi (ARPE-19 cells) or 144 hpi (neurons). Note that while relative and absolute abundances of distinct VZV RNAs differ between cell types, all RNAs included in our annotation are represented. *y* axis denotes coverage range. Canonical (gray), alternative (orange), fusion (dark blue), and putative (red) ncRNAs are shown with canonical CDS regions indicated by wide boxes and UTRs shown as thin boxes. The absence of a CDS region indicates that the respective VZV RNA has an uncertain coding potential. Reiterative repeat regions R1 to R5 and both copies of the OriS are shown as black boxes embedded in the genome track. (B) The scatterplot shows the correlation between VZV transcript abundance in hESC-derived neurons and ARPE-19 cells. Pearson *R*^2^ and *P* value are indicated.

As VZV is capable of infecting diverse cell types including epithelial cells and neurons, we also determined if the VZV transcriptome remains similar between VZV pOka-infected ARPE-19 cells and human embryonic stem cell (hESC)-derived neurons (41, 42). Again, no cell-type-specific novel VZV RNAs or variant VZV RNAs were identified (Fig. 5), indicating that observed differences in VZV RNA expression levels and infectivity in distinct cell types (41, 43) are not due to the presence of cell-type-specific VZV RNAs but are likely driven by host cell factors.

### Overexpression of RNA 43-2 does not impair VZV replication in epithelial cells.

Based on the high relative expression of RNA 43-2, its low protein coding potential, and genomic location, we hypothesized that it may function as an ncRNA involved in regulating the expression of the longer RNA 43-1 (encoding pORF43). RNA 43-1 encodes pORF43 and has previously been designated as an essential gene (44), which putatively encodes the capsid vertex component 1 and is postulated to be important for viral DNA encapsidation from the analogy to HSV UL17 (45). To test this hypothesis, we generated three stable ARPE-19 cell lines expressing RNA 43-2 or an empty vector as control (Fig. S2A and B) and analyzed VZV EMC-1 replication at 48 and 72 hpi by flow cytometry and plaque assay. We did not observe any significant differences in number of VZV-infected cells, number of plaques, or plaque sizes between RNA 43-2-expressing cells and vehicle control cells (Fig. S2C and D). Additionally, RNA 43-2 expression did not significantly reduce expression of RNA 43-1 in VZV-infected cells (*P* = 0.08 by Student’s *t* test) (Fig. S2E).

### Decoding the kinetic class of lytic VZV transcripts.

**FIG 6:**
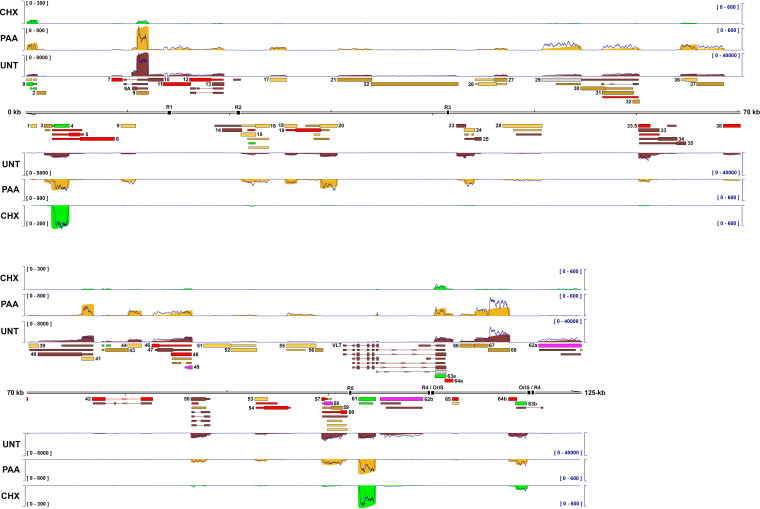
Decoding the kinetic class of VZV transcripts during lytic infection. Coverage plots derived from dRNA-Seq of ARPE-19 cells infected with cell-free VZV EMC-1 and treated with cycloheximide (CHX; green) for 12 h or phosphonoacetic acid (PAA; gold) for 24 h or untreated (UNT; red) for 24 h. Corresponding Illumina RNA-Seq coverage plots for each condition are shown as dark blue line plots. *y* axis denotes coverage range (left side, Nanopore dRNA-Seq; right side, Illumina RNA-Seq). Reannotated VZV genome is shown in the middle track. CDS regions and UTRs are shown as wide and thin boxes, respectively. Reiterative repeat regions R1 to R5 and both copies of the OriS are shown as black boxes embedded in the genome track. Transcripts annotated are colored according to kinetic class: *IE*, green; *TE*, yellow; *EL*, dark gold; *LL*, red; *TL*, dark red; *TA/TL*, purple; *Not classified*, gray. Canonical CDS regions are indicated by wide boxes with UTRs shown as thin boxes. Absence of a CDS region indicates that an RNA has an uncertain coding potential. Reiterative repeat regions R1 to R5 and both copies of the OriS are shown as black boxes embedded in the genome track.

To determine the kinetic class of each lytic VZV RNA, cell-cycle-synchronized ARPE-19 cells were infected with cell-free VZV EMC-1 and cultured for 12 or 24 h in the presence or absence of actinomycin D (ActD; transcription inhibitor), cycloheximide (CHX; translation inhibitor), or phosphonoacetic acid (PAA; inhibitor of the viral DNA polymerase), and viral RNAs were subsequently profiled using dRNA-Seq and RNA-Seq (Fig. 6 and Fig. S3). Transcription of *IE* RNAs is not dependent on *de novo* viral protein production, whereas transcription of *E* RNAs depends on *IE* proteins. *L* RNAs are further subclassified into two kinetic classes, *Leaky-Late* (*LL*) and *True-Late* (*TL*); *LL* RNAs are expressed at very low levels before, and *TL* RNAs exclusively after, viral DNA replication has commenced. To rule out the possibility of contamination introduced through cell-free infections, we also sequenced samples treated with ActD to control for the potential presence of residual background transcripts in the virus preparations and confirmed only minimal amounts of VZV transcripts to be present (Fig. S3).

10.1128/mBio.01568-20.3 FIG S3 Kinetic class of viral RNAs expressed during lytic VZV infection of ARPE-19 cells. (A) Coverage plots derived from Illumina RNA-Seq of ARPE-19 cells infected with cell-free VZV EMC-1 and treated with actinomycin D (ActD; gray), cycloheximide (CHX; green), or phosphonoacetic acid (PAA; gold) or untreated (UNT; red). *y* axis denotes coverage range. Transcripts annotated are colored according to kinetic class: *IE*, green; *TE*, yellow; *EL*, dark gold; *LL*, red; *TL*, dark red; *TA/TL*, purple; *unclassified*, gray. Canonical CDS regions are indicated by wide boxes with UTRs shown as thin boxes. Absence of CDS region indicates that an RNA has an uncertain coding potential. Reiterative repeat regions R1 to R5 and both copies of the OriS are shown as black boxes embedded in the genome track. (B) The inset black hatched box presents a closeup view of the RNA 9 locus. Download FIG S3, TIF file, 0.6 MB. Copyright © 2020 Braspenning et al. 2020 Braspenning et al. This content is distributed under the terms of the Creative Commons Attribution 4.0 International license.

**FIG 7:**
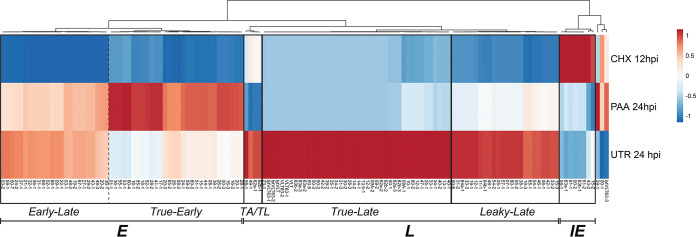
Transcriptional classification of VZV transcripts. The kinetic class of each viral transcript annotated here was derived in isolation by comparing its relative expression in lytically VZV-infected ARPE-19 cells treated with cycloheximide (CHX) or phosphonoacetic acid (PAA) or untreated (UNT). A heatmap was constructed using ClustVis (76), and transcripts were clustered by correlation. This yielded five discrete groupings. The first showed highest relative expression in CHX-treated samples and was annotated as viral *Immediate-Early* (*IE*) RNAs (*n* = 8). The second group showed high relative expressions levels in PAA-treated samples and was annotated as viral *Early* (*E*) RNAs (*n* = 53). Here, two subclusters were formed based on whether high relative expression was sustained (*n* = 24, *Early-Late* [*EL*]) or transient (*n* = 29, *True Late* [*TE*]) in untreated samples. Viral *Leaky Late* (*LL*) RNAs (*n* = 28) were those showing some expression in PAA-treated data sets but higher expression in untreated data sets while viral *True Late* (*TL*) RNAs (*n* = 41) were those that showed high expression only in untreated samples. A final group (*n* = 4) showed detectable yet limited expression in CHX-treated samples, no/minimal expression in PAA-treated samples, and high expression in untreated samples at 24 hpi and has been putatively classified as *Transactivated/True Late* (*TA/TL*).

Given the very high sensitivity of our Illumina RNA-Seq and Nanopore dRNA-Seq analyses, many VZV transcripts were detected under all experimental conditions albeit at vastly different abundancies (Table S3). To classify VZV transcripts into distinct kinetic classes in an unbiased fashion, we determined the relative expression of each of the 136 VZV RNAs in CHX-treated, PAA-treated, or untreated samples and clustered transcripts by average correlation (Fig. 7). In total, 8 transcripts were classified as *IE* and 53 were classified as *E* RNAs, including the experimentally validated transcripts encoding pORF28 and pORF29 (46). A further 28 transcripts were classed as *LL* and 41 transcripts were classed as *TL*, the latter including both RNA 14-1 (pORF14) and VLT (pVLT), both of which have been experimentally confirmed previously (9, 47). Importantly, 4 transcripts were classified as *Transactivated/True-Late* (*TA/TL*)—a putative new designation applied to transcripts that show detectable yet limited expression in CHX-treated samples, no or minimal expression in PAA-treated samples, and high expression in untreated samples at 24 hpi. Three VZV transcripts clustered closely with *IE* RNAs but showed variable expression patterns. One of these, RNA 0-1 (encoding pORF0), shares features with both *IE* and *E* transcripts and was provisionally classed as *IE* based on its relatively high abundance in CHX-treated samples (Table S3), whereas _lyt_VLT63-3 (encoding pORF63) appears to be transactivated during infection and then expressed with *E* kinetics (*TA/E*), and RNA 29-1 showed an expression pattern similar to *E* transcripts. Finally, we also note that *E* transcripts could be classified into two distinct subgroups termed *True-Early* and *Early-Late*, dependent on whether their expression was reduced or consistent in untreated samples compared to PAA-treated samples.

Collectively, our approach showed that two VZV transcripts (RNA 4-1 and RNA 61-1, encoding pORF4 and pORF61, respectively) were expressed at very high levels (accounting for 60% of all VZV transcripts in CHX-treated samples [Table S3]) in the absence of *de novo* protein production and, in agreement with prior studies (23, 25), were classified as *IE* transcripts (Fig. 6 and 7). Six additional transcripts—including both copies of RNA 63-1 (encoding pORF63)—were expressed to high levels in CHX-treated samples relative to other conditions and were also assigned *IE* status, albeit at much lower levels compared to RNA 4-1 and RNA 61-1. pORF4, pORF61, and pORF63 are known transcriptional activators of VZV and canonical *IE* transcripts (22, 23, 25, 48).

### ORF62 transcripts are expressed with *Late* kinetics during lytic VZV infection.

**FIG 8:**
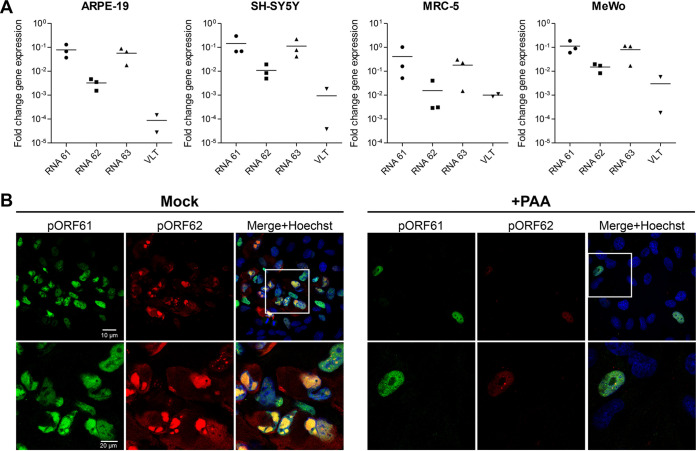
RNA 62 transcripts are expressed with *Late* kinetics during lytic VZV infection. Cell cycle-synchronized human ARPE-19 (epithelial), SH-SY5Y (neuroblastoma), MRC-5 (fibroblast), and MeWo (melanoma) cells were infected with cell-free VZV EMC-1 for 24 h in the presence or absence of PAA. (A) Fold change in expression of transcripts encoding pORF61, pORF62, and pORF63 in PAA-treated samples relative to untreated samples (calibrator) and normalized to glyceraldehyde-3-phosphate dehydrogenase (GAPDH) using the 2^−ΔΔ^*^CT^* method. In 1 of 3 experiments, PAA treatment reduced VLT expression to below detection limit. qPCR primer/probe set directed to the ORF62 CDS recognizes all three transcript variants (RNA 62-1, 62-2, and 62-3). (B) Representative images of lytically VZV-infected ARPE-19 cells stained by immunofluorescence for pORF61 (green) and pORF62 protein (red). Nuclei were counterstained with Hoechst 33342 (blue). Top row, ×400 magnification; bottom rows, ×1,000 magnification.

The VZV gene locus 62 is located within a short-repeat region that is duplicated and sits on either side of the Unique Short (US) region. The gene 62 duplications (62a and 62b) are considered perfect in that even full-length sequence reads align against both copies with an equal match. VZV transcripts RNA 62-1 and RNA 62-2 encode, respectively, the major viral transcriptional activator protein, pORF62, and a predicted N-terminal truncated pORF62 variant. A third transcript (RNA 62-3, *TL*) shows a 3′ truncation that disrupts the CDS through the absence of a stop codon. While this transcript is predicted to encode several putative micro-ORFs, it is not known whether any of these are translated. Surprisingly, our data indicate that following very limited transactivation during initial infection, expression of these transcripts is dependent on *de novo* (viral) protein synthesis and viral DNA replication, thereby classifying these RNA 62 transcripts as *TA/TL*. This contradicts the current classification of RNA62-1 (encoding pORF62) as an *IE* transcript, although we note that this classification was obtained by analogy to the function of its HSV-1 orthologue infected cell polypeptide 4 (ICP4) (49). To confirm and substantiate our findings, we analyzed the impact of viral DNA replication on pORF62-encoding RNA and protein expression in multiple VZV-susceptible cell types at 24 hpi. RT-qPCR analysis showed that PAA treatment was associated with an approximately 10-fold decrease in expression of *IE* transcripts encoding pORF61 and pORF63, consistent with the absence of VZV DNA replication and spread in culture, and a >10,000-fold decrease in expression of *TL*, viral DNA replication-dependent, RNA _lyt_VLT (Fig. 8A). Notably, expression of RNAs encoding pORF62 was more severely affected (∼500-fold reduction) by PAA treatment than that of *IE* RNAs encoding pORF61 and pORF63. Similar results were obtained for VZV strain pOka-infected epithelial ARPE-19 cells, hESC-derived neurons, and lung fibroblast MRC-5 cells (Fig. S4A to C).

10.1128/mBio.01568-20.4 FIG S4 ORF62 transcripts are expressed with *Late* kinetics during VZV infection. (A to C) ARPE-19 cells, hESC-derived neurons, and MRC-5 cells were infected with cell-free VZV strain pOka for 20 to 24 h in the presence or absence of phosphonoformic acid. Fold change in transcript expression in phosphonoformic acid-treated samples relative to untreated samples (calibrator) and normalized to β-actin using the 2^−ΔΔ^*^CT^* method. (D and E) Representative images of VZV strain EMC-1-infected MRC-5 (D) and MeWo (E) cells immunofluorescently stained for pORF61 (green) and pORF62 (red). Nuclei were counterstained with Hoechst stain (blue). Top row, ×400 magnification; bottom rows, ×1,000 magnification. Download FIG S4, TIF file, 1.5 MB. Copyright © 2020 Braspenning et al. 2020 Braspenning et al. This content is distributed under the terms of the Creative Commons Attribution 4.0 International license.

Similarly, PAA treatment severely reduced the abundance and affected the cellular localization of pORF62 in VZV-infected ARPE-19 cells (Fig. 8B). In the absence of PAA, both pORF61 and pORF62 were abundantly expressed in foci of VZV-infected cells, with mostly diffuse nuclear pORF61 staining and pORF62 staining presenting as abundant globular nuclear and diffuse cytoplasmic staining (Fig. 8B, left panels). Consistent with inhibition of VZV replication, no foci of VZV-infected cells were observed in PAA-treated cultures and infected cells were rare. The pORF61 staining pattern in infected cells was comparable between PAA-treated and untreated VZV-infected cells, whereas pORF62 staining intensity was severely reduced and showed weak, mostly diffuse nuclear staining with fewer intensely staining punctae (Fig. 8B, right panels), possibly reflecting incoming pORF62 originating from VZV virions. Identical immunofluorescence (IF) staining results were observed in MRC-5 and melanoma MeWo cells (Fig. S4D and 4E, respectively). Overall, our data demonstrate that RNA 62-1, as well as RNA 62-2 and RNA 62-3, is expressed at low levels prior to viral DNA replication, with robust expression occurring only after viral DNA replication is initiated, consistent with the expression of *LL* but not *IE* transcripts.

## DISCUSSION

Understanding the full coding capacity of a given virus is crucial to understanding its biology. With the advent of new RNA-sequencing methodologies, it has become clear that transcription of herpesvirus genomes is much more complex than previously anticipated. Here, we demonstrate that VZV is no exception and provide a comprehensive reannotation of the VZV transcriptome during lytic infection of human retinal pigment epithelial cells and hESC-derived neurons, incorporating data from two distinct VZV strains. By integrating RNA-Seq, CAGE-Seq, and dRNA-Seq, we have resolved the architecture of the lytic VZV transcriptome. Specifically, we report the TSS and CPAS for all annotated VZV transcripts—including refinement of the 5′ UTRs and 3′ UTRs in RNAs encoding canonical ORFs—and show that VZV further diversifies its transcription through the use of (i) additional TSS and CPAS, (ii) disruption of transcription termination, and (iii) alternative splicing. As a result, several transcript isoforms are expressed from the same locus and fusion RNAs that modify UTRs and CDS of multiple transcripts are produced. Given that 5′ UTR sequences influence the translational efficiency of the downstream CDS (50), alternative UTR usage may provide the virus with an additional mechanism to regulate its protein expression throughout its infectious cycle. Collectively, this study defined 136 polyadenylated VZV RNAs that are expressed during lytic VZV infection, many of which are predicted to increase the diversity of the viral proteome.

Although the VZV genome is considered relatively stable, multiple strains currently cocirculate and recombine (40, 51), potentially influencing the viral transcriptome. However, our comparison of the transcriptional landscape of a VZV clade 1 (strain EMC-1) and a clade 2 (strain pOka) virus revealed that no strain-specific lytic transcript isoforms exist in VZV-infected ARPE-19 cells. However, interstrain differences in repeat variations could nevertheless impact transcription of RNA 11-1 (containing R1), RNA 13.5-1 (R2), RNA 14-1 (R2), RNA 22-1 (R3), RNA 63-2 (R4), RNA 63-3 (R4), and all RNA 59 and RNA 60 isoforms (R5) (35). Similarly, strain-specific polymorphisms may function to extend coding domains such as the N-terminal extended pORF0 (RNA 0-3) in VZV pOka in comparison with other VZV clades (Fig. 4C). Additionally, while previous studies suggested that the VZV transcriptome is generally similar across diverse cell types, none had sufficient resolution or used the methodologies required to disentangle transcript structures (9, 43, 52). Here, we demonstrated that identical transcript isoforms were detected during lytic VZV infection of human retinal pigment epithelial cells and hESC-derived neurons. Thus, while VZV strain-specific polymorphisms and/or cell type may influence viral gene expression, their impact on the lytic VZV transcriptome structure appears to be small.

Many herpesviruses express ncRNAs during lytic and latent infections (53). Previously, we identified the putative dual-function polyadenylated VZV RNA VLT, which encodes a protein expressed during lytic infection but is also functional as an RNA, inhibiting ORF61 RNA expression in overexpression experiments (9). Here, we identified 17 additional polyadenylated VZV RNAs that are predicted to be noncoding. Diverse functions have been attributed to human ncRNAs, including the modification of antisense or overlapping transcription events (54, 55). The generation of functional VZV mutant viruses with impaired expression of identified putative ncRNAs is challenging due to overlap with other viral transcripts. Therefore, we have studied the function of VZV RNA 43-2 by means of RNA 43-2 overexpression followed by VZV superinfection. However, RNA 43-2 did not significantly reduce expression of the overlapping RNA 43-1 transcript nor influence VZV replication. These results most likely reflect the complexity of VZV transcript regulation during lytic infection, as putative ncRNA 43-2 is expressed earlier (*IE* kinetics) and at higher abundance than RNA 43-1 (*E* kinetics) (Fig. 6 and 7 and see also Fig. S2F and G in the supplemental material). Considering the multitude of ncRNAs expressed by other herpesviruses and their crucial roles during infection (53), delineating the functional importance of VZV ncRNAs should be considered a research goal.

Twenty-eight of 136 VZV transcripts are (multiply) spliced. Strikingly, the majority of splicing events occur in a hypercomplex region of the VZV genome encoding both pVLT and pORF61. We have previously shown this locus to be characterized by extensive alternative splicing (9) and here report the presence of multiple transcripts that variously encode a fusion protein of pVLT and pORF63 (pVLT-ORF63) or an N-terminal extended pORF63 (pORF63-N+). The coding potential of pVLT-ORF63 or pORF63-N+ and functional consequences of (and requirement for) these fusion transcripts during reactivation from latency are described in a separate study (78), while their functional role(s) during lytic infection is under investigation. Except for _lyt_VLT63-3, all spliced VZV RNAs used the canonical splice donor (GT) site. The splice donor sites were highly enriched for C/A (−3 position), A (−2), G (−1), and A (+1). All spliced VZV RNAs used the canonical splice acceptor site (AG), often flanked by C (−1) and G/A (+1). Consistent with the use of the cellular splicing machinery to process viral pre-mRNAs, VZV consensus splice donor and acceptor sites closely resemble those of the human transcriptome (56).

Herpesvirus transcripts are traditionally assigned kinetic classes based on their temporal expression pattern and dependence on *de novo* protein synthesis or viral DNA replication. Here, we provide an unbiased transcriptome-wide classification of VZV transcripts during lytic infection of epithelial cells and observed novel patterns of viral RNA expression, designated *TE*, *EL*, and *TA/TL*, in addition to traditional *IE*, *LL*, and *TL* kinetic classes. The data suggest that a subset of the relative expression of *TE* transcripts is reduced following viral DNA replication, whereas this is not the case for *EL* transcripts. Notably, *TE* VZV RNAs are enriched for transcripts encoding proteins involved in viral DNA replication (e.g., RNA 16 variants, RNA 28-1, and RNA 51-1 encoding the viral DNA polymerase processivity factor pORF16, DNA polymerase large subunit pORF28, and origin-binding protein pORF51, respectively), whereas *EL* RNAs encode a more diverse panel of VZV proteins also involved in virion formation and egress (e.g., RNA 31-1 and 37-1 encoding glycoproteins B and H, respectively). *TA/TL* RNAs were detectable at low levels in the absence of *de novo* protein production, but their abundant expression was highly dependent on viral DNA replication. We hypothesize that transcription of *TA/TL* transcripts in CHX-treated samples is due to low-level transactivation by viral tegument proteins delivered from incoming virions. Additionally, our kinetic classification identified that multiple transcripts originating from the same locus could either share the same kinetic class (e.g., RNAs 16-1, 16-2, and 16-3) or be of different kinetic classes (e.g., RNAs 9-1, 9-2, 9-3, and 9A-1) (Table S3 and Fig. S3). While the biological impetus for this is not clear, it seems likely that TSS and CPAS usage are dynamically regulated during lytic infection. For example, RNA 43-1 (*E*, pORF43) and the putative ncRNA 43-2 (*IE*) diverge in CPAS usage and expression kinetics (Fig. 7 and Fig. S3). Similarly, multiple transcripts encoding pORF63 utilizing different TSS are expressed as different temporal classes and various abundancies, with canonical *IE* RNA 63-1 being most abundant, followed by *E* RNA _lyt_VLT63-3 and low quantities of two *TL* RNAs, 63-2 and 63-3 (Fig. 7 and Fig. S3).

Finally, our data provide novel insight into the expression of *IE* transcripts and the role of pORF62 during lytic VZV infection. The most abundantly expressed VZV *IE* transcripts, produced in the absence of new protein synthesis, encode pORF4 and pORF61, which are also the earliest proteins detected during lytic VZV infection (27). Interestingly, prior studies of the RNA 4-1 and RNA 61-1 promoter regions have shown that their efficient transactivation is dependent on pORF62 (57, 58), a major component of the viral tegument (59). However, our data indicate that abundant expression of VZV RNA 62-1 (pORF62), as well as RNAs 62-2 and 62-3, is dependent on viral DNA replication (*TL* and *TA/TL* kinetics), suggesting that tegument-derived pORF62 but not *de novo* pORF62 transactivates RNA 4-1 and RNA 61-1 expression, at least during establishment of the lytic infection cycle. Classification of RNA 62-1 (pORF62) as *TA*/*TL* is also supported by prior observations that a marked increase in pORF62 abundance occurs only after DNA replication has commenced (19). Importantly, our findings do not exclude any of the previously assigned functions of pORF62, most notably its function as a major transcriptional regulator of VZV transcripts (60–62). However, future studies aimed at better understanding the regulation of VZV transcript expression, and the distinct roles of newly produced RNA 62 isoforms, pORF62, and tegument-derived pORF62, are warranted.

In summary, this study describes the detailed analysis of the VZV transcriptome architecture and kinetic classification of viral transcripts in the context of lytic VZV infection. We provide these data as a comprehensive resource that will facilitate functional studies of coding RNAs and their protein products, the role of noncoding RNAs, and the regulation of VZV transcription and translation during lytic infection.

## MATERIALS AND METHODS

### Cells and viruses.

Human retinal pigmented epithelium ARPE-19 cells (American Type Culture Collection [ATCC] CRL-2302) were grown in a 1:1 (vol/vol) mixture of Dulbecco’s Modified Eagle's medium (DMEM) (Lonza) and Ham’s F-12 (Gibco) medium supplemented with 10% heat-inactivated fetal bovine serum (FBS; Lonza) and 0.6 mg/ml l-sodium glutamate (Lonza) or in DMEM/F-12 + GlutaMAX-I (Thermo Fisher Scientific) supplemented with heat-inactivated 8% FBS (Sigma-Aldrich). Human neuroblastoma SH-SY5Y cells were grown in a 1:1 (vol/vol) mixture of Eagle’s Minimum Essential Medium (EMEM) with Earle’s balanced salt solution (EBSS) (Lonza) and Ham’s F-12 (Gibco) medium supplemented with 15% FBS (Lonza), l-sodium glutamate, penicillin-streptomycin, nonessential amino acids (MP Biomedicals), and natrium-bicarbonate (Lonza). MRC-5 human embryonic lung fibroblasts and human melanoma MeWo cells were cultured in DMEM (Lonza), supplemented with 10% FBS, l-sodium glutamate, and penicillin-streptomycin. Human embryonic stem cell (hESC; H9)-derived neural stem cells (NSC) (Thermo Fisher Scientific) were cultured, propagated, and differentiated into neurons as described previously (63). Cell cultures were maintained at 37°C in a humidified CO_2_ incubator. VZV strain pOka (parental Oka) was maintained in, and the cell-free virus was prepared from, ARPE-19 cells as described previously for MRC-5 cells (37). VZV strain EMC-1 is a low-passage-number clinical isolate and was cultured on ARPE-19 cells, and cell-free VZV was extracted as described previously (20, 64). Cell-free EMC-1 was freshly harvested on the day of use and pretreated with DNase I, RNase T_1_, and RNase A (all from Thermo Fisher Scientific) for 30 min at 37°C prior to infection.

### RNA extraction and cDNA synthesis.

ARPE-19 cells were infected with cell-associated VZV EMC-1 by cocultivation of uninfected and VZV EMC-1-infected ARPE-19 cells at an 8:1 cell ratio for 96 h. Alternatively, ARPE-19, SH-SY5Y, MRC-5, and MeWo cells were infected with cell-free VZV EMC-1 for indicated times. Cells were harvested in 1 ml TRIzol (Thermo Fisher Scientific), mixed with 200 μl chloroform, and centrifuged for 15 min at 12,000 × *g* at 4°C. RNA was isolated from the aqueous phase using the RNeasy minikit (Qiagen) according to manufacturer’s instructions, including on-column DNase I treatment, as described previously (65). RNA concentration and integrity were analyzed using a Nanodrop spectrophotometer (Thermo Fisher Scientific), and RNA was subjected to a second round of DNase treatment using the Turbo DNA-free kit (Ambion) according to manufacturer’s instructions. For cDNA synthesis, a maximum of 5 μg RNA was reverse transcribed using Superscript IV reverse transcriptase and oligo(dT) primers (Thermo Fisher Scientific) (RT^+^). As control, the same reaction was performed without reverse transcriptase (RT^−^). Alternatively, RNA was isolated using the FavorPrep blood/cultured cell total RNA minikit (Favorgen Biotech) in combination with the NucleoSpin RNA/DNA buffer set (Macherey-Nagel). DNA was first eluted from the column in 100 μl DNA elution buffer, subsequently the column was treated with recombinant DNase I (20 units/100 μl; Roche Diagnostics) for 30 min at 37°C, and finally RNA was eluted in 50 μl nuclease-free water. RNA was directly treated with Baseline-Zero DNase (2.5 units/50 μl; Epicentre) for 30 min at 37°C. cDNA was synthesized with 12 μl of RNA and anchored oligo(dT)_18_ primer in a 20-μl reaction mixture using the Transcriptor first-strand cDNA synthesis kit at 55°C for 30 min for reverse transcriptase reaction (Roche Diagnostics).

### PCR and sequence analysis.

PCR was performed on RT^+^ and RT^−^ cDNA reaction mixtures using AmpliTaq Gold DNA polymerase (Thermo Fisher Scientific) and primer pairs corresponding to each newly identified VZV transcript (see Table S5 in the supplemental material). Primers were directed to the predicted 5′ and 3′ ends of each transcript so that newly identified transcripts were completely amplified from the 5′ to the 3′ end. For ORF9 variants and ORF48, additional reverse primers were used to confirm splice junctions (Table S5). PCR amplification was performed as follows: initial denaturation at 95°C for 10 min, followed by 40 cycles of alternating denaturation (30 s, 95°C), primer annealing (30 s at the appropriate temperature) (Table S5), and subsequently primer extension (1 min/1,000 bp, 72°C) (Table S5). A final extension step of 10 min at 72°C was included. To amplify ORF13.5, each deoxynucleoside triphosphate (dNTP), including equimolar amounts of dGTP and 7-deaza-GTP (New England Biolabs), was used at a concentration of 200 μM (66). PCR amplification of ORF13.5 was performed as follows: initial touchup PCR from 58°C to 70°C using a transcript-specific forward primer and an anchored primer on the poly(A) tail. Subsequently, seminested PCR was performed using the same forward primer and a reverse primer within the transcript using standard PCR protocol. Amplicons were purified from gel using the QIAquick gel extraction kit (Qiagen) and sequenced using the BigDye v3.1 cycle sequencing kit (Applied Biosciences) with corresponding forward and reverse primers on the ABI Prism 3130 XL genetic analyzer.

10.1128/mBio.01568-20.9 TABLE S5 Database of primers used in this study. Download Table S5, XLSX file, 0.02 MB. Copyright © 2020 Braspenning et al. 2020 Braspenning et al. This content is distributed under the terms of the Creative Commons Attribution 4.0 International license.

### Plasmid construction and generation of stable cell lines.

The RNA 43-2 transcript sequence (77775 to 78619 excluding the intron at 77869 to 78149; strain Dumas, NC_001348.1) was amplified using cDNA from VZV EMC-1-infected ARPE-19 cells and primers NheI_ORF43-5_Fw and XhoI_ORF43.5_Rv (Table S5). Amplicon was digested with NheI and XhoI and cloned into pcDNA3.1. Three independent batches of ARPE-19 cells were transfected with either pcDNA3.1/empty or pcDNA3.1/RNA 43-2 using polyethylenimine (PEI). After 2 days, cells were incubated with 1 mg/ml Geneticin and cultured for at least 3 weeks to select for transfected cells. Subsequently, DNA was isolated using the QIAamp DNA minikit according to manufacturer’s instructions and the presence of RNA 43-2 DNA was confirmed by PCR. Next, RNA was isolated as described above, and expression of RNA 43-2 was confirmed by RT-PCR.

### Flow cytometry.

ARPE-19 cells stably transfected with pcDNA3.1/empty or pcDNA3.1/RNA 43-2 were plated 1 day prior to infection in a 48-well plate. Cells were infected with cell-free VZV EMC-1 (multiplicity of infection [MOI] = 0.01), harvested at 48 hpi or 72 hpi, fixed and permeabilized with BD Cytofix/Cytoperm, stained for VZV glycoprotein E (gE) (MAB8612; Millipore) in BD PermWash, and labeled with secondary allophycocyanin (APC)-conjugated goat anti-mouse Ig antibody (BD Biosciences). The frequency of VZV-infected (i.e., APC-positive) cells was measured on a BD FACSLyric flow cytometer and analyzed using FlowJo software (BD Biosciences).

### Plaque assay.

Confluent monolayers of stable pcDNA3.1-empty or pcDNA3.1-RNA 43-2 cells grown in a 12-well plate were infected with 2,000 PFU/well VZV EMC-1. At 72 h postinfection, plates were washed and fixed with 4% paraformaldehyde (PFA) in PBS. Subsequently plates were permeabilized using 0.1% Triton X-100 in PBS for 10 min, blocked with 5% normal goat serum in PBS-0.05% Tween 20 (PBS-T) for 30 min, and incubated with mouse anti-VZV gE antibody (MAB8612) diluted in PBS-T containing 0.1% bovine serum albumin (BSA) for 1 h at room temperature. Cells were washed with PBS-T, incubated for 1 h with polyclonal rabbit anti-mouse Ig antibody (Dako) in PBS-T + 0.1% BSA, washed, and stained with Alexa Fluor 488 (AF488)-conjugated goat anti-rabbit Ig (H+L) antibody (Thermo Fisher) in PBS-T. Plates were measured using the Immunospot S6 Ultimate UV image analyzer, and plaque size was determined using Immunospot software (Cellular Technology Limited).

### Kinetic class of VZV transcripts.

ARPE-19 cells were synchronized in the cell cycle using a double thymidine block approach (67). Briefly, ARPE-19 cells were seeded at semiconfluence in 12-well plates, and on the next day medium was replaced with growth medium containing 2 mM thymidine (Sigma-Aldrich). After 24 h, medium was replaced with normal growth medium for 8 h, after which medium was changed back to thymidine-containing medium. Thirty minutes prior to infection, cells were released from thymidine by replacing the medium with regular culture medium. Cells were infected with freshly harvested cell-free VZV EMC-1 using spin-inoculation for 15 min at 1,000 × *g* (MOI after spin-inoculation = 0.1 to 0.2). Cells were incubated for 45 min at 37°C, after which the inoculum was replaced with fresh medium, medium with 10 μg/ml actinomycin D (ActD; Sigma-Aldrich), medium with 50 μg/ml cycloheximide (CHX; C4859; Sigma-Aldrich), or medium with 400 μg/ml phosphonoacetic acid (PAA; Sigma-Aldrich). Infected cells were harvested in 500 μl TRIzol at 12 hpi (ActD and CHX) or 24 hpi (untreated and PAA) for RNA extraction. Alternatively, ARPE-19 cells, MRC-5 cells (1 × 10^5^ cells/well on a 24-well plate), and hESC-derived neurons (1 × 10^5^ cells/well on a 24-well plate as NSC and differentiated for 18 days) were infected with cell-free VZV pOka (20 μl of 4 × 10^4^ PFU/ml) in the presence or absence of phosphonoformic acid (200 μg/ml) (Sigma-Aldrich) for 1 h, the inoculum was replaced with fresh medium with phosphonoformic acid (200 μg/ml), and cultures were maintained for 24 h.

### Quantitative PCR analysis.

Quantitative TaqMan real-time PCR (qPCR) was performed in duplicate on RT^−^ and RT^+^ cDNA using 4× TaqMan Fast Advanced master mix (Applied Biosystems) on a 7500 TaqMan PCR system. Primer-probe sets directed to CDS regions of RNAs 61, 62, and 63 and VLT have been described previously (9, 68), and those directed to ORF43 are described in Table S5 in the supplemental material. Alternatively, cDNAs were subjected to qPCR using KOD SYBR qPCR mix (Toyobo) in the StepOnePlus real-time PCR system (Thermo Fisher Scientific) (1 μl of cDNA per 10-μl reaction mixture). All primer sets used for SYBR green chemistry (Table S5) were first confirmed for the amplification rate (98 to 100%) using 10 to 10^6^ copies (10-fold dilution) of pOka-BAC genome or VLT plasmid (9) and the lack of nonspecific amplification using water. The qPCR program is as follows; 95°C for 2 min (1 cycle), 95°C for 10 s and 60°C for 15 s (40 cycles), and 60 to 95°C for a dissociation curve analysis. Data are presented as relative VZV level to cellular beta-actin defined as 2 − (threshold cycle [*C_T_*] value VZV transcript − *C_T_* value beta-actin).

### Northern blot analysis.

Biotinylated probe directed to VZV ORF64 (nucleotides 111646 to 111936 in reference strain Dumas, NC_001348.1) was generated using the MAXIscript kit (Ambion; Thermo Fisher Scientific) according to the manufacturer’s instructions. Northern blot analysis was performed using the NorthernMax-Gly kit (Ambion). In brief, 7.5 μg total RNA extracted from mock- and VZV EMC-1-infected ARPE-19 cells was separated by electrophoresis on a 1% agarose gel and transferred to a nylon membrane (Brightstart-Plus; Ambion). Membranes were incubated for 15 min at 80°C to cross-link RNA, prehybridized in ULTRAhyb buffer for 30 min at 68°C, and hybridized with biotinylated ORF64-specific probe (0.1 nM diluted in ULTRAhyb buffer) overnight at 68°C. Probe binding was visualized using the AP chemiluminescent blotting kit (KPL) and ChemiDoc MP imaging system (Bio-Rad).

### Immunofluorescence staining.

ARPE-19 cells were plated on glass coverslips in 24-well plates 1 day prior to infection. Cells were inoculated with freshly harvested cell-free VZV EMC-1 in medium with or without 400 μg/ml PAA and incubated for 24 h. Infected cells were fixed with 4% PFA, permeabilized for 10 min with 0.1% Triton X-100 in PBS, blocked with 5% goat serum diluted in 0.2% gelatin-PBS solution, and incubated on 30-μl 0.2% gelatin-PBS droplets containing primary antibody overnight at 4°C. The following primary antibodies were used: anti-pORF61 antibody (1:1,000, gift from P. Kinchington) and monoclonal mouse anti-pORF62 antibody (1:200) (20). Cells were washed 3 times with 0.2% gelatin-PBS and incubated for 1 h at room temperature with secondary antibodies diluted in 0.2% gelatin-PBS. The following secondary antibodies were used: AF488-conjugated goat anti-rabbit Ig (H+L) antibody (1:500; Thermo Fisher) and AF594-conjugated goat anti-mouse Ig (H+L) antibody (1:500; Thermo Fisher). Cells were washed once with 0.2% gelatin-PBS, washed once with PBS, incubated with a 1:1,000 dilution of Hoechst 33342 (Life Technologies, 20 mM) in PBS for 5 min, washed with PBS, and mounted using Prolong Gold antifade mounting medium (Thermo Fisher). Stained cells were analyzed using a Zeiss LSM 700 confocal laser scanning microscope (Zeiss) with a magnification of ×400 or ×1,000. Photoshop CC 2019 software (Adobe) was used to adjust brightness and contrast.

### Illumina RNA sequencing and analysis.

Stranded RNA libraries were prepared from poly(A)-selected RNA using the NEBNext Ultra II Directional RNA library prep kit for Illumina (New England Biolabs) and sequenced using a NextSeq 550. Sequence reads were trimmed using TrimGalore (https://www.bioinformatics.babraham.ac.uk/projects/trim_galore/) (–paired –length 30 –quality 30) and aligned against the VZV reference genome (strain Dumas, NC_001348.1) using BBMAP (https://sourceforge.net/projects/bbmap/) with postalignment processing performed using SAMtools (69) and BEDTools (70) to generate BEDGRAPH and BED12 files. Candidate CPAS were identified using ContextMap2 (71) (-aligner_name bowtie –polyA –strand-specific) with sequence reads aligned to VZV Dumas under default parameters by BWA (72).

### Cap Analysis Gene Expression (CAGE) sequencing (CAGE-Seq) and analysis.

Two biological replicates of total RNA were extracted from ARPE-19 cells infected with VZV pOka for 96 h and CAGE-Seq libraries prepared by DNAform (Yokohama, Japan) and subsequently sequenced using an Illumina NextSeq 550, as previously described (31). Resulting sequence reads were trimmed (–length 30 –q 30 –clip_R1 1) using TrimGalore prior to alignment against the VZV Dumas genome using BBMAP. Postalignment processing was performed using SAMtools and BEDTools. TSS were identified using the HOMER findPeaks module (-style tss -localSize 100 -size 15). Only TSS present in both biological replicates were retained for analysis.

### Nanopore direct RNA sequencing.

For each biological sample, up to 1,000 ng of poly(A) RNA was isolated from up to 50 μg of total RNA using the Dynabeads mRNA purification kit (Invitrogen; catalog no. 61006). Isolated poly(A) RNA was subsequently spiked with 0.3 μl of a synthetic Enolase 2 (ENO2) calibration RNA (Oxford Nanopore Technologies Ltd.), and dRNA-Seq libraries were prepared as described previously (10). Sequencing was performed on a MinION MkIb using R9.4.1 (rev D) flow cells (Oxford Nanopore Technologies Ltd.) for 18 to 44 h (one library per flowcell) and yielded between 720,000 and 1,290,000 reads per data set (Table S4). Raw fast5 data sets were then basecalled using Guppy v3.2.2 (-f FLO-MIN106 -k SQK-RNA002) with only reads in the pass folder used for subsequent analyses. Sequence reads were aligned against the VZV Dumas genome, using MiniMap2 (73) (-ax splice -k14 -uf –secondary=no), with subsequent parsing through SAMtools and BEDTools. Here, sequence reads were filtered to retain only primary alignments (alignment flag 0 [top strand] or 16 [bottom strand]).

### Splice junction correction in dRNA-Seq alignments.

Illumina-assisted correction of splice junctions in RNA-Seq data was performed using FLAIR v1.3 (30) in a stranded manner. Briefly, Illumina reads aligning to the VZV Dumas genome were split according to orientation and mapping strand (-f83 & -f163 [forward] and -f99 & -f147 [reverse]) and used to produce strand-specific junction files that were filtered to remove junctions supported by less than 50 Illumina reads. Direct RNA-Seq reads were similarly aligned to the adenovirus type 5 (Ad5) genome and separated according to orientation (-F4095 [forward] and -f16 [reverse]) prior to correction using the FLAIR correct module (default parameters). FLAIR-corrected alignments were used for all subsequent downstream analyses.

### TSS and CPAS identification in dRNA-Seq data.

TSS and CPAS were identified by parsing SAM files to BED12 files in a strand-specific manner using BEDTools and then truncating each aligned sequence read to its 5′ or 3′ termini for TSS and CPAS identification, respectively. Peak regions containing TSS and CPAS were identified using the HOMER findpeaks module (-o auto -style tss) using a –localSize of 100 and 500 and –size of 15 and 50 for TSS and CPAS, respectively. TSS peaks were compared against Illumina annotated splice sites to identify and remove peak artifacts derived from local alignment errors around splice junctions. Thirty-eight putative TSS identified in the dRNA-Seq data set alone were flagged as artifacts and removed. Each of these TSS mapped precisely to a splice acceptor within a spliced RNA, and closer inspection of the reads showed these to result from local alignment processes (10).

### Generating RNA abundance counts from dRNA-Seq data.

Using the updated VZV strain Dumas annotation presented here, we generated a transcriptome database by parsing our GFF3 file to a BED12 file using the gff3ToGenePred and genePredtoBED functions within UCSCutils (https://github.com/itsvenu/UCSC-Utils-Download) and subsequently extracting a fasta sequence for each annotated RNA using the getfasta function within BEDTools. dRNA-Seq reads were then aligned against the transcriptome database using parameters optimized for transcriptome-level alignment (minimap2 -ax map-ont -p 0.99). RNA abundance counts were generated by counting alignments against a given RNA only if the alignment 5′ end was located within the first 50 nt of the RNA and the alignment was not marked as supplementary. Transcript per million (TPM) counts (Fig. 3) were generated by dividing the RNA abundance count for a given transcript by the total number of sequence reads (one read = one RNA) present in the data set and subsequently multiplying by 1 million. Note that where raw abundance counts were <3, these counts were zeroed out to minimize possible artifact introduced by low-level noise.

### *In silico* prediction of coding potential.

CPC 2.0 (36) was used to examine the coding potential of all VZV RNAs defined in this study (Table S2). Note that RNAs were excluded from CPC 2.0 analysis and defined as putatively noncoding if no proteins greater than 50 amino acids in length were encoded.

### Data visualization.

Figures associated with this study were generated using the R packages Gviz (74) and GenomicRanges (75). Heatmaps were generated by uploading TPM counts (Table S3) to ClustVis (76) with grouping of transcripts performed using the average correlation function.

### Statistical analysis.

Statistical analyses were performed where appropriate using GraphPad Prism 5 software with specific tests indicated in the figure legends.

### Data availability.

All sequencing data sets associated with this study are available via the European Nucleotide Archive under the accession number PRJEB38829. Analyzed data sets generated as part of this study, including a database of transcripts, BED12 alignment files, and GFF3 files describing our VZV annotation, are freely available at https://github.com/DepledgeLab/vzv-2.0.
