# Supplementary figures and images for: Decoding the Architecture of the Varicella-Zoster Virus Transcriptome
Source: mBio. 2020 Oct 6;11(5):e01568-20. doi: 10.1128/mBio.01568-20 (PMC7542360; doi:10.1128/mBio.01568-20)

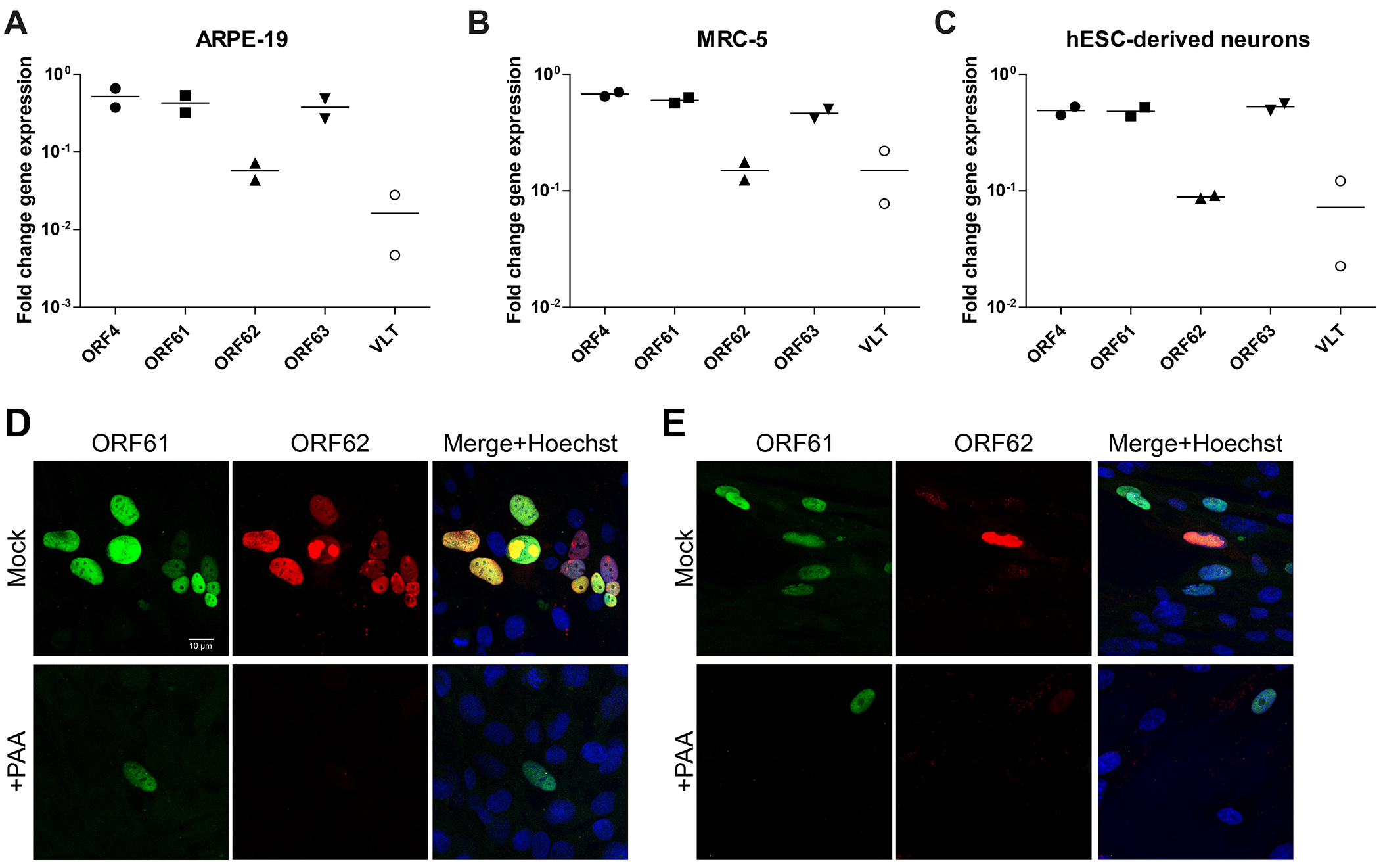

Supplement: FIG S4 [file mBio.01568-20-sf004.tif]
